# Supplementary figures and images for: Predictive reward-prediction errors of climbing fiber inputs integrate modular reinforcement learning with supervised learning
Source: PLoS Comput Biol. 2025 Mar 17;21(3):e1012899. doi: 10.1371/journal.pcbi.1012899 (PMC11957396; doi:10.1371/journal.pcbi.1012899)

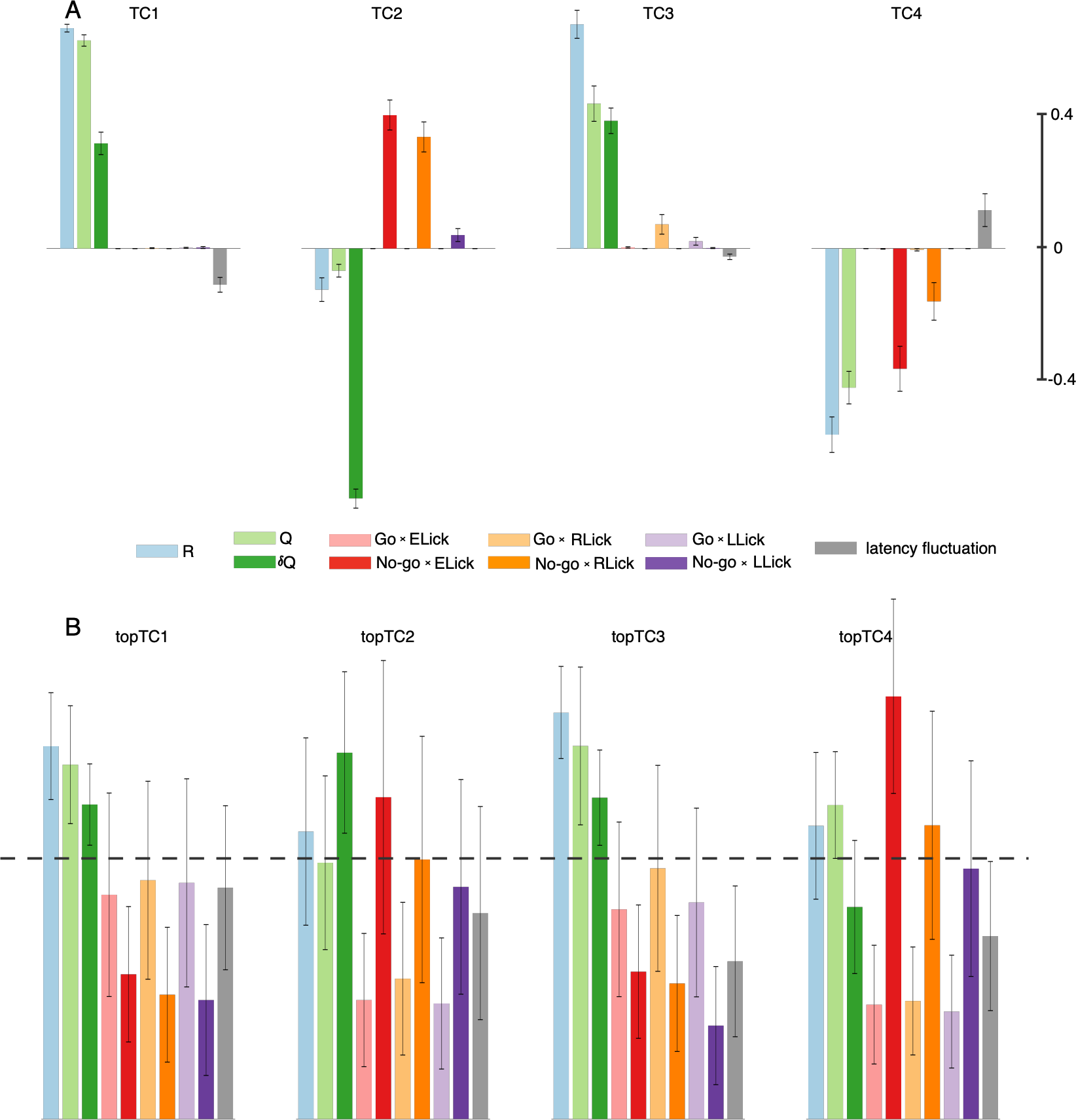

Supplement: S1 Fig — A: sCCA of TC score and behavior variables were conducted for individual animals. Bar and lines indicate the averaged and std of CCA coefficients across 17 animals. B: we sampled the top 300 neurons for each TC at each learning stage, then removed overlapping ones (see [26] for details). As a result, we selected 2,096 neurons (termed “topTC neurons”) and individually conducted PLSR of their TC score with the behavior variables. Bar and lines indicate the averaged and std of VIP score for topTC1-4 neurons. The horizontal dashed line indicates VIP score = 1. Color convention is the same as Fig 2. (TIFF) [file pcbi.1012899.s001.tiff]

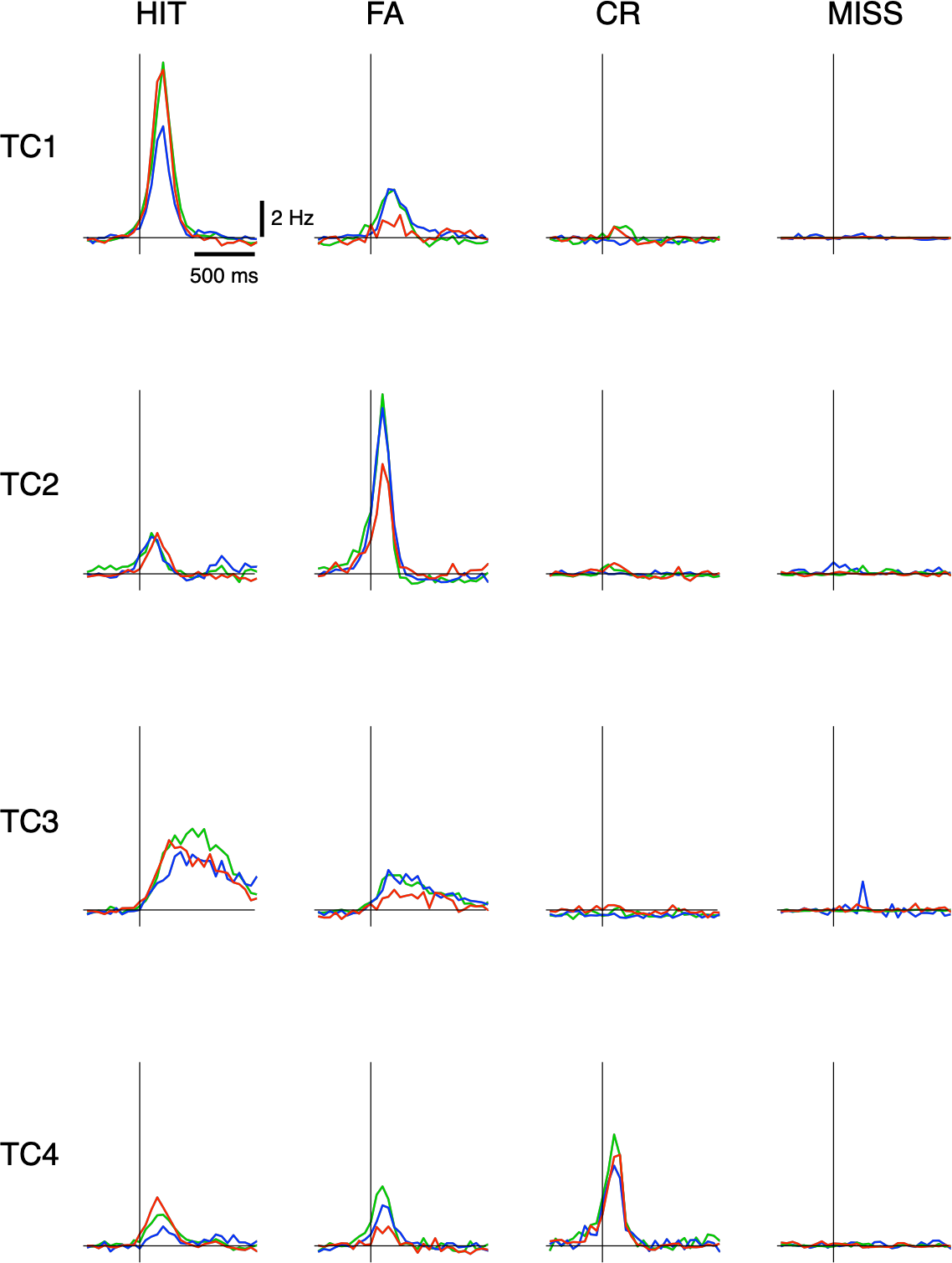

Supplement: S2 Fig — Blue, green and red traces are for 1st, 2nd and 3rd learning stages, respectively. (TIFF) [file pcbi.1012899.s002.tiff]

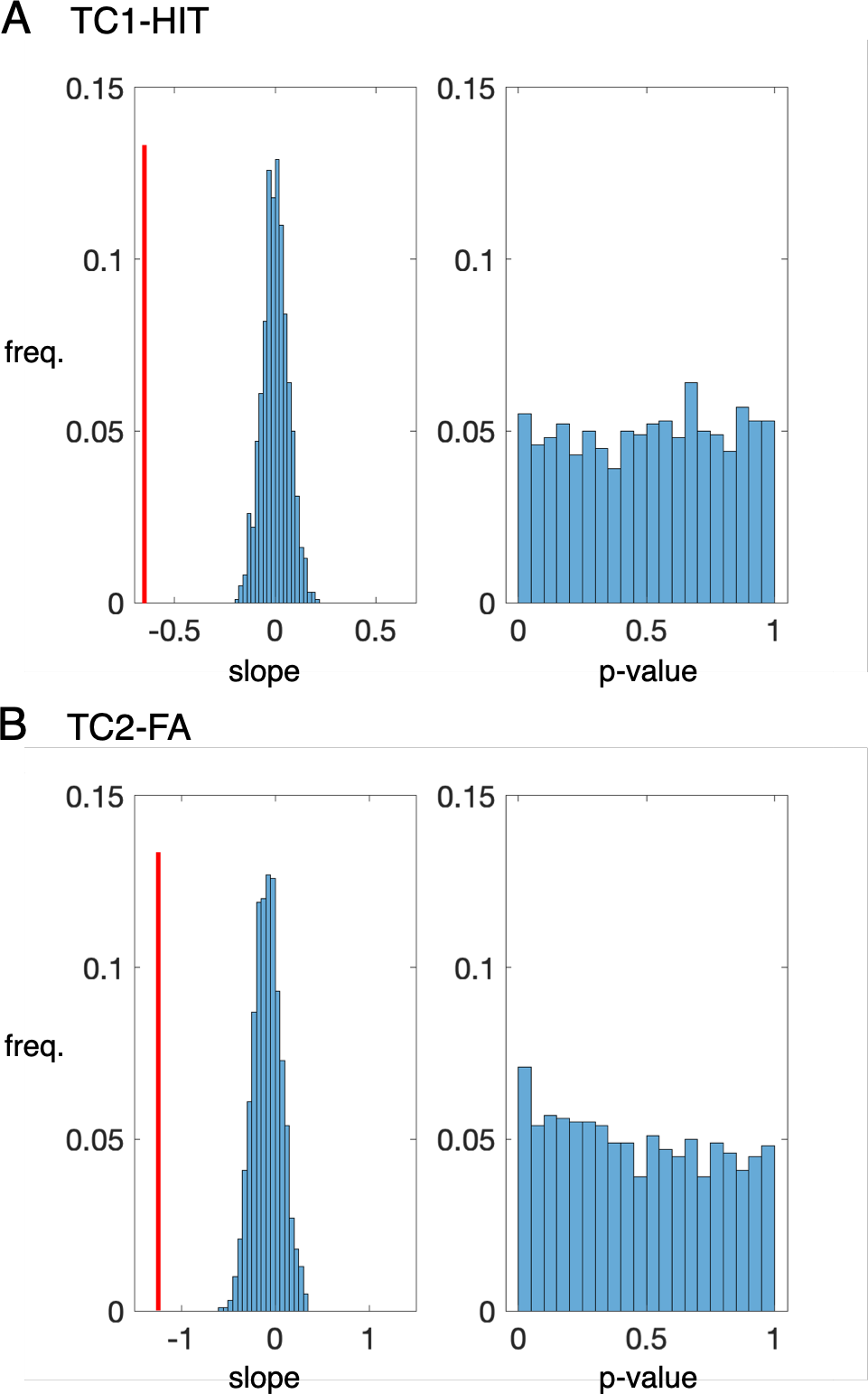

Supplement: S3 Fig — A: For each animal, we shuffled the pairs of TC1 score vs. reward-prediction errors δQ in HIT trials and conducted the regression analysis similar to Fig 4C. The bar-plots indicated frequency of the slope (left) and p-value (right) of the regression for 1000 shuffled times. B: similar to A but for TC2 score vs. δQ in FA trials. Note that, for the original data, the slope of regression was -0.65 for TC1-HIT and -1.24 for TC2-FA (vertical red lines) with p-value < 0.00001 (reported in Fig 4C&D). (TIFF) [file pcbi.1012899.s003.tiff]

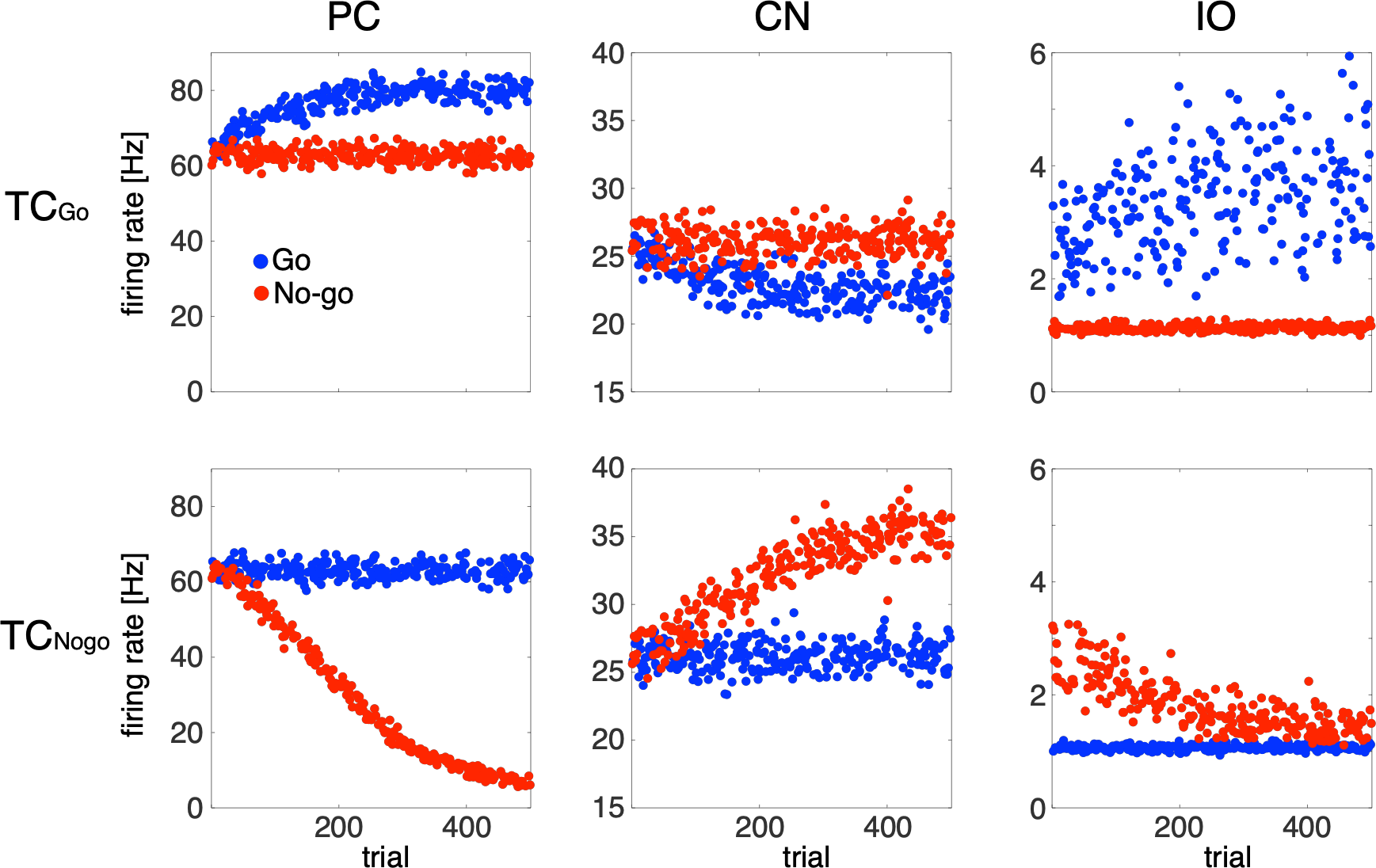

Supplement: S4 Fig — (TIFF) [file pcbi.1012899.s004.tiff]

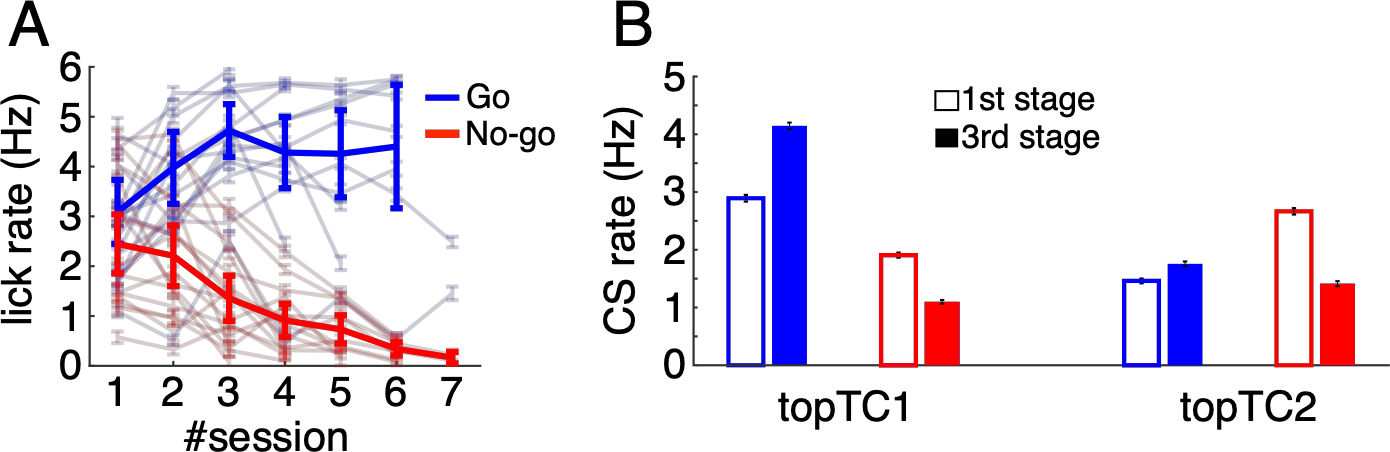

Supplement: S5 Fig — The thick lines represent the average lick rate across all animals. B:Summary of top TC neurons in the real data. Bars with error bars indicate the mean ± s.e.m of CS rate during Go (blue bars) and No-go (red bars) trials for topTC1 and topTC2 neurons (refer to S1B Fig for neuron sampling) in the first stage (open bars, fraction correct < 0.6) and the third stage (filled bars, fraction correct > 0.8). The differences in CS rate between the 1st and 3rd stages were significant (p < 0.0001) for all conditions. Note that both lick rate and CS rate were measured within 0-0.5 s after cue onset. (TIFF) [file pcbi.1012899.s005.tiff]

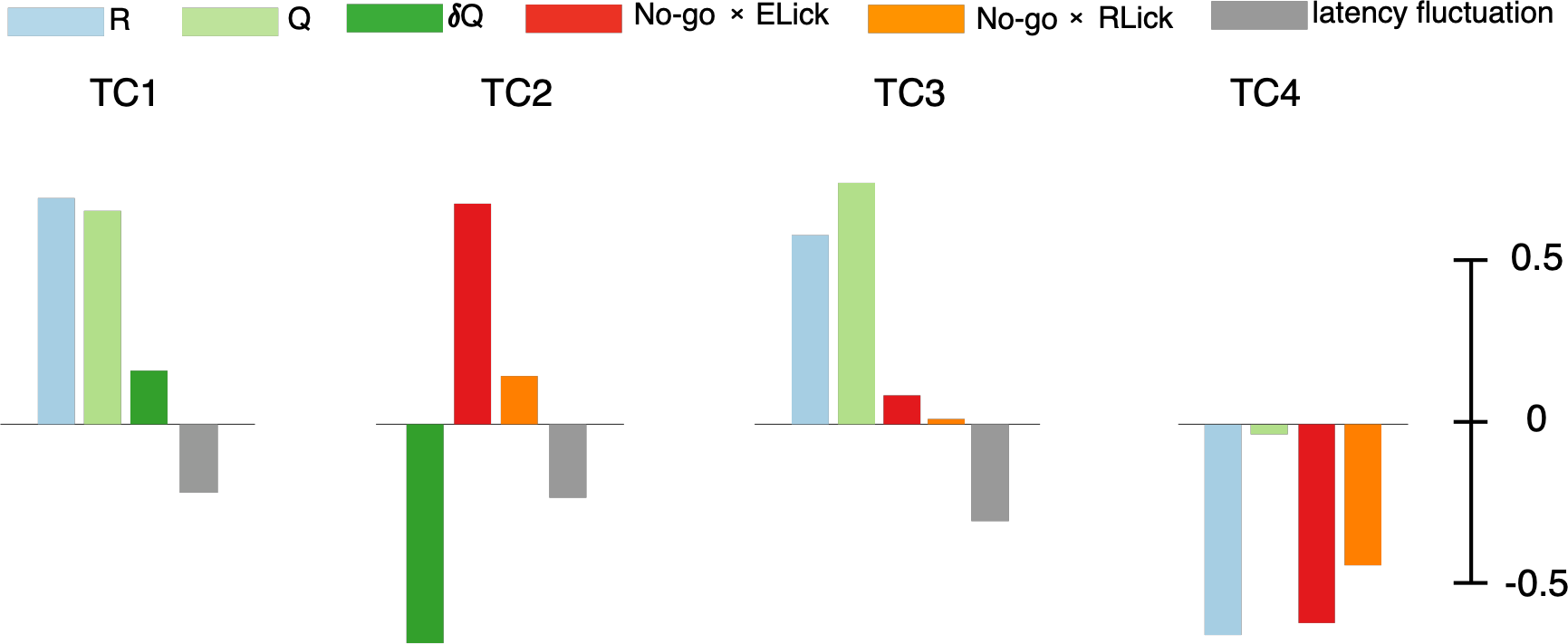

Supplement: S6 Fig — (TIFF) [file pcbi.1012899.s006.tiff]

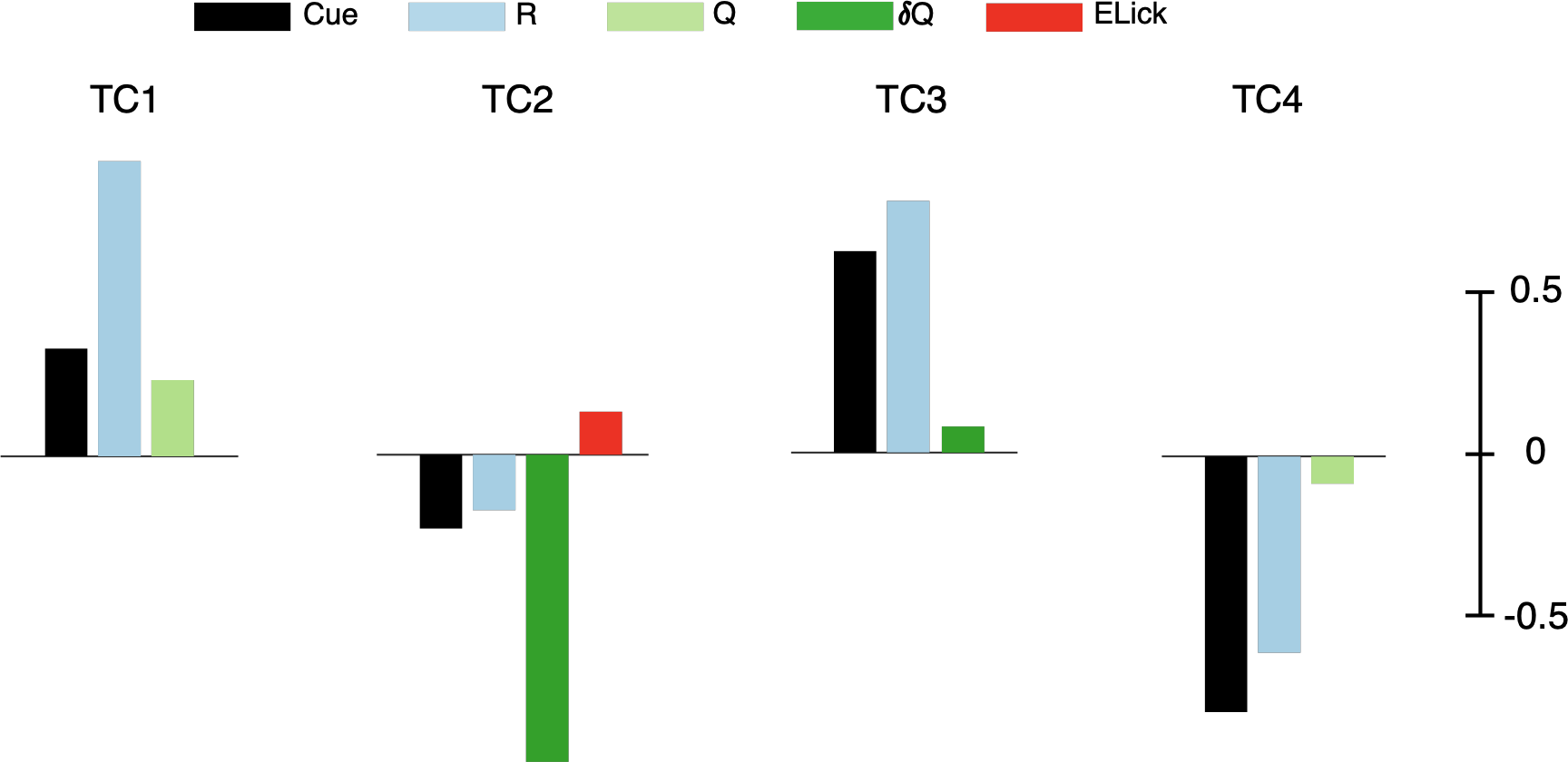

Supplement: S7 Fig — In these analyses, the auditory cue (Cue=1 for Go and Cue=0 for No-go cues) was made independent from motor (licking) variables, resulting in a total of 8 exploratory variables (Cue, R, Q, δQ, ELick, RLick, LLick and lick latency fluctuation). sCCA selected 5 exploratory variables, with only a small positive correlation between TC2 and early lick count. Color convention is the same as Fig 2 with a new black column corresponding to the auditory cue. (TIFF) [file pcbi.1012899.s007.tiff]

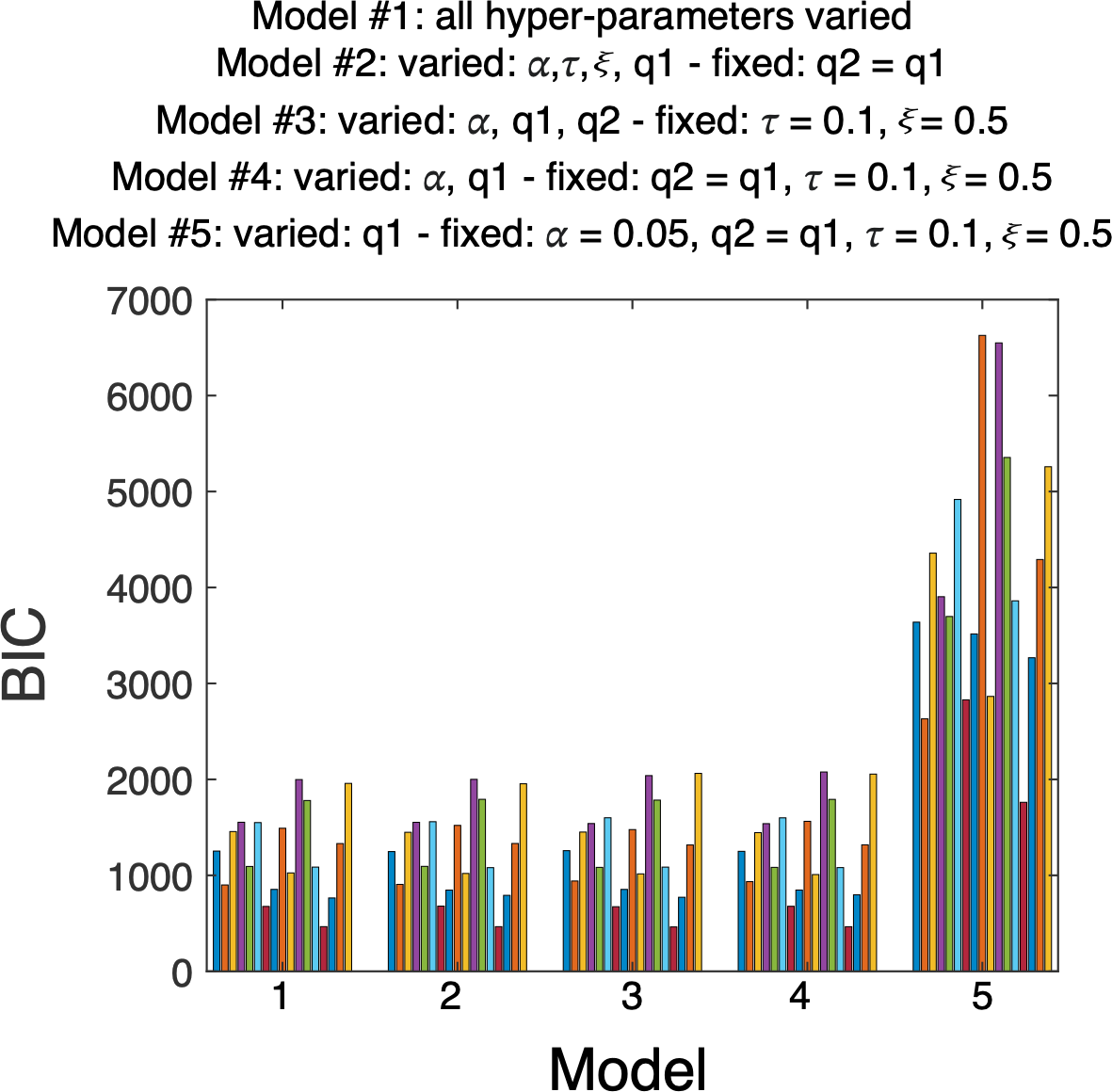

Supplement: S8 Fig — The total BIC score was 21,235; 21,291; 21,414; 21,532 and 69,325 for Model #1-5, respectively. (TIFF) [file pcbi.1012899.s008.tiff]
