# Supplementary material for: Predictive reward-prediction errors of climbing fiber inputs integrate modular reinforcement learning with supervised learning
Source: PLoS Comput Biol. 2025 Mar 17;21(3):e1012899. doi: 10.1371/journal.pcbi.1012899 (PMC11957396; doi:10.1371/journal.pcbi.1012899)
Supplement: Attachment — Submitted filename: response_to_reviewer_v1.4.docx [file pcbi.1012899.s010.docx]

Dear Dr. Abigail Morrison and Dr. Lyle Graham,

We sincerely appreciate your thoughtful handling of our manuscript and thank the two reviewers for their valuable comments and constructive feedback. Their insights have significantly improved the clarity and overall quality of our work. We have thoroughly addressed the suggestions from the reviewers as well as other journal requirements, with the revisions tracked in the updated manuscript. Additionally, we confirm that Figure 1A was drawn by hand and does not involve any copyright issues. We hope that the current version meets the publication standards of *PLOS Computational Biology*.

Sincerely yours,

Huu Hoang, Shinichiro Tsutsumi, Masanori Matsuzaki, Masanobu Kano, Keisuke Toyama, Kazuo Kitamura, Mitsuo Kawato

===

Reviewer #1: The manuscript describes a combined experimental and computational study of reinforcement learning in the cerebellum. The research integrates a Q-learning model with experimental spike detection, Tensor Component Analysis (TCA) of the neuronal activity, correlation analyses of the neuronal activity against reinforcement learning and sensorimotor control variables, and a spiking neural network model of the olivo-cerebellar system. This is excellent work and beautifully presented – I recommend publication in PLOS Computational Biology subject to minor revisions.

We appreciate the reviewer's positive feedback and valuable suggestions, which we have carefully addressed in the revised manuscript.

Minor comments:

1. In the neural network model, the lick rate is modelled as a sigmoid function of the combined spike rate of cerebellar nucleus (CN) neurons, with decreasing lick rates for increasing CN spike rates (and a maximum lick rate at a CN spike rate of zero). This differs from many other cerebellar models (e.g. Medina et al. J Neurosci. 2001) that assume movements are triggered by increased output from the CN. It would be good to discuss the neural circuitry downstream of the CN that could implement this mapping of decreased (including zero) CN output on increased lick rates.

Thank you for this important suggestion. Experimental studies in eye-blink conditioning (ten Brinke et al., Cell Reports, 2015; ten Brinke et al., Elife, 2017) and cerebellar models (Medina et al., J. Neurosci., 2001) have demonstrated that suppression of simple spikes (SSs), leading to increased output from the cerebellar nuclei (CN), drives movements. However, Purkinje cells influence both excitatory and inhibitory neurons within the CN (de Zeeuw and Berrebi, 1996), meaning decreased CN output could also drive movements. Specifically, if the downstream circuitry of Purkinje cells includes substantial inhibitory connections—comprising inhibitory CN neurons and motor neurons—then learning of a specific motor action may primarily occur through increased SS activity or decreased CN activity. This mechanism has been identified in at least two paradigms, vestibulo-ocular reflex adaptation (Voges et al., J Physiol. 2017) and reflexive whisker protraction (Romano et al., eLife, 2018), as well as in computational models (Badura et al., Sci. Rep., 2016). Aligned with this mechanism, our model includes inhibitory CN neurons that modulate the electrical coupling between IO neurons. We have discussed this divergence in cerebellar network mechanisms in the revised manuscript (Lines 559-574, Page 23).

In our model, the lick rate is represented as a sigmoid function of the combined spike rate of CN neurons. For simplicity, the model assumes increasing lick rates with decreasing CN spike rates (discussed above), with the maximum lick rate occurring at low CN spike rates (including zero). However, in our simulations, CN neurons exhibit spontaneous firing rates of approximately 25 Hz (Figure S4), ensuring that CN activity never drops to zero. We have mentioned this point in the revised manuscript (Lines 561-563, Page 23).

2. Although this is already briefly mentioned in the discussion, the network model doesn’t represent a few important anatomical features, in particular, the convergence of many Purkinje cells on CN neurons. It would be good to discuss the expected effect of these features in more detail.

We completely agree with the reviewer regarding this point. The convergence of multiple Purkinje cells onto CN neurons is a critical feature of cerebellar anatomy that likely affects the integration of inhibitory signals in the network. This anatomical feature could potentially lead to an increased level of synchrony between different Purkinje cell inputs, modulating the firing patterns of CN neurons in a way that reflects more complex integration of timing control signals (Person and Raman, Nature, 2011; Zobeiri and Cullen, Nat. Comm. 2024; Gruver et al., Nat. Comm. 2024). Additionally, the convergence could influence how the cerebellum accounts for noise and variability in the output, since multiple Purkinje cells might help refine the signal sent to CN neurons, smoothing out the response (Steuber and Jaeger, Neur. Netw. 2012). In our model, we simulated only the lick rates, not the timing of licks, and did not account for noise. Therefore, the convergence of multiple PC inputs onto CN neurons was not considered. We discussed this point in the revised manuscript (Lines 574-586, Pages 23-24).

3. The neural network only represents TC1 and TC2, but not TC3 and TC4, although they are also (positively / negatively) correlated with the reward and licking. Would it be possible to speculate about a neural network (without additional simulations) that includes TC3 and TC4?

Thank you very much for this very important suggestion to include TC3 and TC4. We now realize that our description regarding this point was erroneous. We should state TC1 & TC3 when we described TC1, and similarly TC2 & TC4 when we described TC2. We thoroughly edited the descriptions for Figs 5, 6, and 7 (Pages 13-18). In more detail, in a previous study, we analyzed CS-triggered lick responses of TC3 neurons during Go trials across three response windows: early lick (0–0.5 s), reward lick (0.5–2 s), and succeeding lick (2–4 s) (Figure 6 – figure supplement 2A, Hoang et al., 2023, shown here for reference). Lick responses were largest during the reward window, with a balanced distribution of negative and positive values, suggesting that TC3 is equally involved in sensory feedback and motor control of reward-related licks. For TC4 neurons, slightly increased CS firing with learning—particularly in AldC compartment 6+—supported the hypothesis that TC4 enhances the suppression of early licks following the No-go cue, resulting in more correct-rejection (CR) trials and fewer false alarm (FA) trials (Figure 6 – figure supplement 2B, Hoang et al., 2023). This idea was further supported by both the spike-triggered lick responses from the previous study (Figure 6 – figure supplement 2C, Hoang et al., 2023) and the regression analysis in the current study (Fig. 4). Additionally, as discussed in the manuscript, we hypothesize that TC2 transitions into TC4 due to their strong overlap in temporal profile, spatial distribution, and No-go cue specificity. In this context, we propose that TC4 and TC2 share the same underlying neural network and mechanisms in the Go/No-go task. Likewise, the neural network underlying TC3 may resemble that of TC1, with the key distinction being that TC3 was activated specifically during the reward delivery period. This suggests that TC3’s climbing fibers transmit distinct or multiplexed information to modulate licking behaviors during this phase.

Figure 6 – figure supplement 2, Hoang et al., 2023: Possible functions of TC3 and TC4.

4. Line 155: “CSs in HIT trials (n = 3,788) were large and distributed across the entire medial hemisphere at the initial learning stage, but later on (2nd and 3rd stages), they became stronger and compartmentally focused on positive zones.” – Figure 2 A shows that positive zones (in particular, 5+ and 5a+) already stand out in the initial learning stage.

You’re right. We have revised the manuscript accordingly (Lines 175-178, Page 8).

5. Figure 2 A also shows that the CS activity increases during learning (from stage 1 to stage 3) in CR trials?

You’re right. The CS activity, particularly in zone 6+, slightly increases in CR trials during learning. We have updated the manuscript to reflect this (Lines 180-181, Page 8).

6. Line 180: “We found that neurons in the lateral hemisphere were associated with Q and dQ, but not the reward R per se (Fig 2B).” – Figure 2 B indicates that the lateral zone 7+ is also strongly associated with R.

You’re right. We have revised the manuscript accordingly (Lines 204-205, Page 9).

7. Line 334: “two groups of neurons, corresponding to TC1 and TC2, operating within a modular architecture where each group independently processes Go and No-go cues” - strictly speaking, only the climbing fibre input represents Go and No-go cues independently, the mossy fibre input to the TC1 and TC2 groups seems to be shared.

Sensory input for the Go and No-go cues was transmitted to Purkinje cells via mossy fibers (MFs), with Go and No-go cues being relayed through two separate groups of granule cells (GrC_Go_ and GrC_Nogo_). However, TC1 & TC3 and TC2 & TC4 neurons received shared mossy input, which is represented in the model by the equal connection of GrC_Go_ and GrC_Nogo_ to both PC_Go_ and PC_Nogo_. We have revised the manuscript to make this point clearer (Lines 396-397, Page 17).

8. Please check the grammar of a few sentences, e.g. line 119 “As temporal evolution …”, line 576 “while the probability …”.

We have corrected the grammar of those sentences (Lines 137-139, Page 6 and Lines 642-545, Page 26) and throughout the manuscript.

9. Line 394: “IO firings” --> IO spiking or IO firing.

We have revised the manuscript accordingly (Lines 427-436, Page 18).

Reviewer #2: This is a nice study from Dr Hoang and colleagues examining the information content of complex spike Ca responses across large populations of Purkinje neurons during a go-no-go task. The data have been previously published, but the study here extends the analyses employed in those previous studies to examine the whether the observed patterns of CS activity can be used in a Q-learning reinforcement learning algorithm. Further, they advance a straightforward computational model that accounts for the distinct modules they observe with CS tuning. I think the study represents a nice advance. I do not have any substantive scientific suggestions. However, I think it is important for the authors to explicitly spell out in the main text what is new and what has previously been published. Some of the figure panels are identical (or practically identical, with different color lookup tables applied) to previously published work for the same group (e.g. Hoang et al., 2023). In reading that manuscript there is significant overlap with the present study, but nevertheless the present study is distinct and worthy of separate publication. That said, a more explicit enumeration of the distinctions between the two studies will be a service to the readership.

We appreciate the reviewer for taking the time to read our two papers and provide thoughtful feedback. We share the reviewer's concern about the overlap between the two papers, as both analyze the same dataset. However, as the reviewer pointed out, while the previous paper focused on the dynamic organization of climbing fiber inputs during the learning of Go/No-go tasks, the aim of the present paper is to investigate the potential role of climbing fiber inputs in reinforcement learning algorithms. To achieve that, we extend our analyses in three significant ways: (1) by employing a Q-learning algorithm to model licking behavior and compute reinforcement learning variables, such as reward-prediction errors; (2) by conducting trial-by-trial regression analyses to link climbing fiber activity to both reward- and sensorimotor-related variables; and (3) by developing a cerebellar neural network model that incorporates bidirectional synaptic plasticity to simulate how Purkinje cells generate context-specific motor commands based on reward-prediction errors. As suggested by the reviewer, we have revised the Introduction to summarize the results from the previous paper and highlight the new results and analyses presented in this study (Lines 63-92, Pages 4-5).

Regarding Figures 2A and 4A in the current paper, which are technically identical to Figures 2A-D and 3 in Hoang et al., 2023, respectively, we felt it was important to include them here for clarity and to help readers follow the arguments presented. However, to reduce redundancy, we replotted Figure 2A, replacing heatmaps with line plots for PSTHs. We also emphasized in the Results that these figures are essentially the same as those in our previous study (Lines 166-168, Page 8 and Lines 236-237, Page 10).

Minor
1. In lines 156-159 I would suggest that the authors call the responses CSs instead of PSTHs.

We have revised the manuscript according to this suggestion (Lines 172-182, Page 8).

2. In Hoang et al., 2023 the authors describe ‘error’ responsive CSs and reward-responsive CSs. I found this language helpful to classify different types of responses. Here the description of CS tuning does not use these descriptions, even for CSs that are negatively correlated with reward, or responsive to false alarms. It would be helpful to better match the descriptions in the two papers or to provide an explanation as to why the previous nomenclature no longer holds.

In the previous study (Hoang et al., 2023), "error" response was measured as the number of licks in the 0–0.5 s window following No-go cues, while "reward" response was defined as licks in the 0.5–2 s window following Go cues. Please note that the previous study did not explicitly define "reward" variables; rather, it only measured licking activity within the "reward" window. In this study, these responses are redefined as two sensorimotor-control variables: ELick x No-Go and RLick x Go, respectively. We believe this updated nomenclature more effectively distinguishes reward-related variables from sensorimotor control variables, enabling a clearer separation of these aspects compared to the previous study. Additionally, this nomenclature facilitates systematic regression analyses performed in this study, such as examining the relationship between CS responses and six sensorimotor-control variables (ELick, RLick, and LLick under both Go and No-go conditions). A note has been added to the revised manuscript to emphasize this distinction (Lines 487-492, Page 20).
